# Longitudinal association between fitness and metabolic syndrome: a population-based study over 29 years follow-up

**DOI:** 10.1186/s12889-024-18448-3

**Published:** 2024-04-06

**Authors:** Johannes Wiemann, Janina Krell-Roesch, Alexander Woll, Klaus Boes

**Affiliations:** 1 Institute of Sports and Preventive Medicine, Saarland University, D-66123 Saarbrücken, Germany; 2 Institute of Sports and Sports Science, Karlsruhe Institute of Technology, D-76131 Karlsruhe, Germany

**Keywords:** Fitness, Exercise, Metabolic syndrome, Health, Longitudinal study

## Abstract

**Objectives:**

To examine the longitudinal associations between fitness and metabolic syndrome (MetS) in community-dwelling adults over 29 years of follow-up.

**Design:**

Ongoing, population-based cohort study of adults aged ≥ 33 years at baseline residing in the city of Bad Schönborn, Germany.

**Methods:**

The sample comprised 89 persons (41 females; mean age 40.1 years at baseline) who participated at baseline (in the year 1992) and 29-years follow-up (in the year 2021). Fitness (predictor variable) was assessed using 15 standardized and validated tests that measured strength, gross motor coordination, mobility/ flexibility and cardiorespiratory fitness/ endurance, and a z-transformed fitness score was calculated for analysis. MetS (outcome of interest) was assessed through five criteria related to waist circumference, blood glucose, HDL cholesterol, triglycerides, and blood pressure, and a sum score was created for analysis. We ran partial correlations to examine the association between fitness score at baseline and MetS score at 29-years follow-up, adjusted for age, sex, socio-economic status, smoking status, sleep quality, and physical activity engagement in minutes/ week.

**Results:**

A higher fitness score at baseline was significantly associated with a lower MetS score indicative of better metabolic health at 29-years follow-up (*r*=-0.29; *p* = 0.011). These associations were present in participants aged ≤ 40 years (*r*=-0.33; *p* = 0.025) as well as those aged > 40 years (*r*=-0.43; *p* = 0.045).

**Conclusions:**

Fitness may be a predictor of longitudinal metabolic health, and potentially also mediates previously reported longitudinal associations between physical activity and metabolic health. More research is needed to confirm these observations, and to also explore underlying mechanisms.

**Supplementary Information:**

The online version contains supplementary material available at 10.1186/s12889-024-18448-3.

## Introduction

Metabolic syndrome (MetS) is an umbrella term for different conditions that are partly interrelated, including high blood pressure, disturbed lipid metabolism, and insulin resistance [1]. MetS has high prevalence and incidence in both western industrial nations and emerging countries [2]. This increase is mainly due to lifestyle behavior changes in recent decades, i.e., increased caloric intake and access to high-caloric food, as well as decreased physical activity and increased sedentary behavior [2]. To this end, we and others have reported that higher physical activity is longitudinally associated with a decreased risk of MetS in community-dwelling adults [3, 4]. Furthermore, intervention studies have also shown beneficial effects of lifestyle or physical activity programs on metabolic health (e.g. [5, 6]).

Similar to physical activity, fitness is also regarded as a predictor of health, and higher fitness levels have been associated with lower mortality [7] and morbidity [8], decreased coronary heart disease or cardiovascular diseases [9], cancer [10], dementia [11], and depressive symptoms [12], amongst others. In addition, a growing body of research has shown that adults with higher fitness levels also have a significantly lower risk of having or developing MetS [13, 14]. Furthermore, individuals with MetS may experience significant health improvements through aerobic or resistance training, either alone or combined [15]. It has also been postulated that an increase in physical activity alone may not lead to significant improvements in physical health, but health status may be improved if an increase in physical activity is concomitant with an increase in fitness level. Fitness may thus be regarded as a mediator on the association between physical activity and health status [16].

However, most studies published on the associations between fitness and MetS thus far, focused on cardiorespiratory fitness [17, 18] or selected fitness variables, e.g., handgrip strength as predictors [19, 20]. Less is known, as to whether general fitness is related to metabolic health. Also, to the best of our knowledge, there are no studies published to date that examined the associations between flexibility or gross motor coordination with metabolic health in adults. Furthermore, most studies have relatively shorter follow-up periods of up to 15 or 20 years. To date, only few population-based studies on fitness and health exist that have long follow-up periods of ≥ 25 years, such as the Harvard Alumni Health Study [21], the Framingham (Heart) Study [22], the Copenhagen City Heart Study [23], the Korean Genome and Epidemiology Study [24], or the Tokyo Oldest Old Survey on Total Health [25].

To address these research gaps, the aim of our study was to investigate the longitudinal associations between fitness (total fitness score as well as score in different fitness components, i.e., muscular strength, gross motor coordination, mobility/ flexibility and cardiorespiratory fitness/ endurance) and MetS in community-dwelling adults over a follow-up period of 29 years. We hypothesized that a higher baseline fitness score and higher individual fitness component scores would be associated with a lower MetS score at follow-up.

## Methods

This research was conducted in the setting of the “Gesundheit zum Mitmachen“ study, an ongoing, population-based prospective cohort study of middle-aged and older adults residing in the city of Bad Schönborn in southwestern Germany. So far, the study had six measurement points, i.e., in the years of 1992, 1997, 2002, 2010, 2015, and 2021. Design and methodology have been described in detail elsewhere [16] and are only briefly outlined here. In the first year of the study, the initial sample was drawn by stratified randomized sampling from the population register, using age strata of 35, 40, 45, 50 and 55 years. Of 857 individuals invited, a total of 480 persons participated in the initial assessments (56% response rate) [16]. If a person took part in the study at any measurement point, he or she was automatically invited again for the subsequent measurement. In addition, the sample at each measurement point was augmented with further individuals between 33 and 37 years of age and residing in Bad Schönborn who had not participated in the study before. The representativeness of the 1992 baseline sample was examined by comparison with the municipal statistics and by using data from a dropout analysis through a telephone survey of non-participants. Limitations concerning the representativeness of the 1992 study sample existed only with regard to the percentage of foreign-born citizens who were underrepresented in the study [16]. Overall, a total of 1090 individuals have participated in the study thus far. The number of participants for each measurement point were as follows: 1992: N = 480; 1997: N = 456 (327 participated at all prior measurements, 129 newly enrolled); 2002: N = 428 (213 participated at all prior measurements, 112 newly enrolled, 103 participated at one prior measurement); 2010: N = 310 (129 participated at all prior measurements, 181 participated at one or more prior measurements); 2015: N = 422 (74 participated at all prior measurements, 238 newly enrolled, 110 participated at one or more prior measurements); 2021: N = 430 (44 participated at all prior measurements, 131 newly enrolled, 255 participated at one or more prior measurements).

For the current analysis, we considered only individuals who participated in the study in the years of 1992 and 2021 (*N* = 89; 41 females). The study was conducted according to the guidelines of the Declaration of Helsinki. The ethics committee of the Karlsruhe Institute of Technology (KIT), Germany, approved the study. All participants provided written informed content for participation in the study. To avoid disclosing personal information, all participants’ names have been encoded with numbers.

Fitness status was assessed by 15 validated test items (the reader is referred to [16, 26] for a detailed description of the fitness assessment methodology). Participants performed all tests under the supervision of trained sports science students in a single session on one day by following a standardized test protocol.

Cardiorespiratory fitness/ endurance was assessed by a bicycle ergometer test, and VO_2_max in liters/ minute was estimated (with higher VO_2_max reflecting higher endurance). Strength was assessed by 4 tests, i.e., number of push-ups performed in 40 s (with higher number corresponding to higher strength), number of sit-ups performed in 40 s (with higher number corresponding to higher strength), handgrip strength left and right (in kg; with more kg reflecting higher strength), and a jump-and-reach-test (in cm; with more cm reflecting higher strength). Flexibility/ mobility was assessed by 5 tests, i.e., a sit-and-reach test (in cm), trunk side bending (in cm), shoulder neck mobility (in cm), with more cm reflecting higher flexibility/ mobility; and hamstring and rectus femoris muscle extensibility (in cm; with less cm reflecting higher flexibility/ mobility). Gross motor coordination was assessed by a 5-item test battery including standing on one leg with closed eyes, standing on one leg while moving the second leg in circles, and three test items involving balls, i.e., throwing the ball to the wall, performing a 360° body rotation and subsequent catching of the ball; throwing the ball into the air, performing a 360° body rotation and subsequent catching of the ball; and grasping the ball between the knees five times. For each gross motor coordination test, the performance was classified by a staff member in one of three categories, i.e., well done, done, or failed. With regard to strength and coordination as well as the sit-and-reach test, the best of two attempts was taken. All other fitness tests were only carried out once by each study participant.

All test items were carefully selected and quality criteria (i.e., objectivity, reliability and validity) was examined and confirmed in cooperation with investigators from the UKK Institute, Finland [26]. All test variables were z-transformed, with the mean value of the age group of 33- to 36-year-old males in 1992 as the reference (corresponding to a score of 100 and a standard deviation of 10). For statistical analysis, we calculated an overall fitness score by considering all 15 fitness test items. This score was only calculated if a participant had valid data for at least 50% of the tests from gross motor coordination, mobility/ flexibility and strength. In the score, the four fitness components (i.e., cardiorespiratory fitness/ endurance, strength, flexibility/ mobility and gross motor coordination) were weighted equally, and thus each component accounts for 25% of the fitness score. Similarly, we calculated scores for each fitness component, by including each of the individual test items with equal weight. For example, to create the strength score, each of the four test items (i.e., number of push-ups performed in 40 s, number of sit-ups performed in 40 s, handgrip strength left and right, and jump-and-reach-test) contributes 25% to the overall strength score of a participant.

MetS criteria were assessed through a medical examination of all study participants by a licensed physician and corroborated by self-reported information provided by participants through a questionnaire, e.g., on medication intake. We used the 2001 National Cholesterol Education Program (NCEP) Adult Treatment Panel III (ATP III) definition of MetS for this study [27], i.e., waist circumference ≥ 102 cm (for males) or ≥ 88 cm (for females); blood glucose > 110 mg/dl or diagnosed diabetes; HDL cholesterol < 40 mg/dl for males or < 50 mg/dl for females; triglycerides ≥ 150 mg/dl; and blood pressure ≥ 130/85 mmHg. For statistical analysis, we created a MetS score, i.e., one point was assigned for each diagnostic criterion fulfilled; the score thus had a possible range of 0 to 5 points.

With regard to anthropometric measurements, we determined body weight and height of participants using a calibrated scale on a solid surface and a mobile stadiometer from Seca®. Participants were asked to remove clothes and shoes before the measurement. When determining body height, the distance between the foot sole and the vertex was measured with participants in an upright posture. Body mass index (weight [kg]/height [m]^2^) was calculated, and waist circumference was measured using a tape measure when participants were in upright standing position.

Blood pressure was measured during the medical examination. After at least five minutes of rest in seated position, blood pressure was determined with an automatic measuring device (M500 intellisense, Omron K.K., Kyoto, Japan). Blood pressure was measured on the left upper arm in a seated position with the back supported. Systolic and diastolic blood pressure were expressed in mmHg.

Blood was drawn from each study participant by a licensed physician, and samples were analyzed on site using the TASCOM Simplex TAS101 (TASCOM Co. Ltd., Anyang, South Korea). We determined long-term blood glucose value HbA1c, total cholesterol, HDL cholesterol and triglycerides. LDL cholesterol was calculated using the Friedewald formula. On each study day, an internal quality control check of the device was carried out. There is a high correlation between the TASCOM system used in this study with laboratory devices such as the Hitachi 7020 clinical chemistry system for all blood parameters assessed in this study (Total Cholesterol: r²=0.9952; Triglycerides: r²=0.9973; HDL-cholesterol: r²=0.9883; HbA1c: r²=0.9894) [28]. We assessed HbA1c value instead of blood glucose since the fasting state of a participant does not need to be considered. It has been shown that blood lipids can also be determined without fasting, as there is no clinically significant difference compared to fasting values [29].

In addition to age and sex, all participants were asked about their socio-economic status (SES). To this end, a stratum index was formed from information provided by participants on the level of education and the occupational group to which they belonged [16]. For statistical analysis, we used four SES groups, i.e., lower class (1), lower middle class (2), upper middle class (3) and upper class (4). We also considered self-reported information provided by participants through validated questionnaires about smoking status, sleep quality and physical activity behavior as potential confounders for our analyses. Smoking status was assessed through two items (Are you a smoker? yes/ no; Are you a previous smoker? yes/ no). Sleep quality was assessed using a five-point Likert scale ranging from 1 (not at all true) to 5 (totally true) in relation to the statement “I usually sleep well”; and participants who responded “1 = not at all true” and “2 = slightly true” were considered as having impaired sleep quality. Furthermore, participants estimated the minutes per week and weeks per year at which they engaged in sports-related physical activity (such as jogging or swimming) and general physical activity (such as walking and cycling for transportation purpose). Validity and reliability of the questions on physical activity for use in epidemiological studies have been demonstrated [30].

We carried out descriptive analysis, with categorical variables expressed through number (N) and percentage (%), and continuous variables expressed through means (M) and standard deviations (SD). To examine the associations between fitness score at baseline (1992) and MetS score at 29-years follow-up (2021), we calculated partial correlations adjusted for age, sex, SES, smoking status, sleep quality, and physical activity engagement in minutes/ week. The confounding factors were collected at baseline. We also carried out partial correlation analyses stratified by sex (males and females) and age group (≤ 40 years and > 40 years). The significance level was set at *p* ≤ 0.05 for all tests. Statistical analyses were conducted using SPSS Statistics (IBM, Armonk, USA, version 28 for Windows).

## Results

A total of 89 participants (41 females, 46%) were included in the analysis. The mean age was 40.1 (SD 5.7) years at baseline. Table 1 provides an overview of demographic characteristics of the sample at baseline (year 1992), stratified by sex.

**Table 1:** Demographic characteristics of study sample at baseline and follow-up (*N* = 89)

	Baseline		Follow-up	
	Females (*N* = 41)	Males (*N* = 48)	Females (*N* = 41)	Males (*N* = 48)
Age in years; mean (SD)	38.7 (4.5)	41.2 (6.3)	68.3 (4.5)	70.3 (6.0)
BMI; mean (SD)	23.3 (2.9)	25.6 (2.8)	25.1 (3.3)	26.7 (4.0)
Fitness score (z-transformed); mean (SD)	93.9 (5.3)	97.4 (6.4)	79.1 (5.5)	82.5 (10.0)
CR fitness/ endurance (z-transformed); mean (SD)	84.7 (5.7)	97.3 (8.3)	81.6 (4.5)	81.0 (18.4)
Strength (z-transformed); mean (SD)	83.7 (6.2)	98.4 (7.4)	54.5 (7.5)	75.0 (10.5)
Flexibility/ mobility (z-transformed); mean (SD)	106.7 (7.2)	98.0 (9.9)	100.9 (8.5)	93.3 (10.7)
GM coordination (z-transformed); mean (SD)	97.9 (11.5)	96.6 (9.2)	80.0 (9.2)	80.3 (10.7)
MetS score; mean (SD)	N.a.	N.a.	1.9 (1.0)	1.8 (1.1)
Waist circumference in cm; mean (SD)	77.9 (8.5)	91.4 (9.3)	86.4 (8.9)	100.1 (10.8)
Blood glucose in mg/dl; mean (SD)	86.7 (14.6)	95.3 (21.9)	N.a.	N.a.
HDL-Cholesterol in mg/dl; mean (SD)	65.0 (16.7)	59.2 (13.9)	77.2 (19.0)	65.5 (20.5)
Systolic BP in mmHg; mean (SD)	118.3 (11.5)	130.7 (12.4)	149.5 (16.7)	147.0 (18.8)
Diastolic BP in mmHg; mean (SD)	73.3 (8.3)	78.8 (8.1)	85.7 (8.3)	87.9 (9.5)
HbA1c in %; mean (SD)	N.a.	N.a.	5.6 (0.5)	5.6 (0.4)
Triglycerides in mg/dl; mean (SD)	N.a.	N.a.	169.2 (109.2)	141.6 (82.5)
SES score; mean (SD)	2.9 (0.7)	3.1 (0.9)	2.5 (0.9)	2.6 (1.0)
Sports-related PA in min/week; mean (SD)	107.7 (112.9)	113.9 (96.6)	230.5 (205.4)	182.1 (184.6)
General PA in min/ week; mean (SD)	244.6 (179.7)	343.9 (309.7)	416.0 (253.9)	480.1 (333.8)
Smoker; N (%)	14 (34.1)	21 (44.7)	5 (14.3)	17 (37.8)
Impaired SQ; N (%)	6 (14.6)	7 (15.2)	18 (47.4)	15 (31.9)

*SD, standard deviation; BMI, body mass index; CR fitness, cardiorespiratory fitness; GM coordination, gross motor coordination; MetS, metabolic syndrome, possible range 0–5; SES, socio-economic status, possible range 1–4; PA, self-reported physical activity; SQ, sleep quality; N.a., data not available*

Association between fitness at baseline and MetS score at follow-up.

After adjusting for sex, age, SES, smoking and sleep quality, a higher fitness score at baseline was significantly associated with a lower MetS score at 29-years follow-up (*r* = -0.29; *p* = 0.011). Additional adjustment for sports-related and general physical activity slightly increased the correlation (*r* = -0.30; *p* = 0.009). However, there was no significant correlation between fitness score at baseline and MetS score at 29-years-follow-up when only adjusting for age, sex and SES (*r* = -0.21; *p* = 0.059; supplementary Table 2). In analyses stratified by sex and adjusted for age, SES, smoking, sleep quality, sports-related PA and general PA, there were no significant associations between baseline fitness and follow-up MetS score in either males (*r* = -0.31; *p* = 0.068) or females (*r* = -0.31; *p* = 0.084). Furthermore, in analyses stratified by age group and adjusted for age, SES, smoking, sleep quality, sports-related PA and general PA, there were significant correlations between fitness score at baseline and MetS score at 29-years-follow-up for participants aged ≤ 40 years at baseline (*r* = -0.33; *p* = 0.025) as well as those > 40 years of age at baseline (*r* = -0.43; *p* = 0.045).

Association between fitness components at baseline and MetS score at follow-up.

After adjusting for sex, age, SES, smoking, sleep quality and physical activity, higher strength (*r* = -0.30; *p* = 0.012) and gross motor coordination (*r* = -0.28; *p* = 0.017) at baseline were significantly associated with a lower MetS score at 29-years follow-up. There were no associations between cardiorespiratory fitness/ endurance (*r* = -0.09; *p* = 0.493) or flexibility (*r* = -0.044; *p* = 0.709) at baseline with MetS score at 29-years follow-up (supplementary Table 2).

## Discussion

We observed a significant association between higher baseline fitness and lower MetS score reflecting better metabolic health at 29-years follow-up in community-dwelling adults, after adjusting for age, sex, SES, smoking status, sleep quality, and physical activity engagement. Interestingly, there was a lack of a significant correlation between fitness score at baseline and MetS score at follow-up when only adjusting for age, sex, and SES. We can speculate from the literature that physical activity is independently associated with both, fitness and MetS. Thus, any correlation between fitness and MetS should be adjusted for physical activity. Given the impact of physical activity on both fitness and MetS, it is plausible that we did not see any association between fitness and MetS, when we only adjusted for age, sex and SES, and did not consider physical activity. Furthermore, the associations between higher baseline fitness and higher MetS score at 29-years follow-up were present in persons aged ≤ 40 years at baseline as well as > 40 years. In line with previous literature [13, 14], this provides further evidence that fitness may be a predictor of longitudinal metabolic health outcomes in adults.

When examining the associations between different fitness components at baseline with MetS at follow-up, we observed that strength and gross motor coordination, but not cardiorespiratory fitness/ endurance or flexibility at baseline were associated with a lower MetS score at 29-years follow-up. The lack of an association between cardiorespiratory fitness and MetS is unexpected, but may be due to methodological issues as follows: In 1992, we used a bicycle ergometer test to estimate VO_2_max as indicator of cardiorespiratory fitness. However, several participants had problems to adjust to riding a stationary bicycle ergometer. Thus, from the next measurement wave in 1997 onwards, the bicycle ergometer test was replaced by the 2-km walking test, and time to complete the 2 km (in seconds) was documented. The test was well received by participants. For the purpose of this study, we ran secondary analyses by calculating correlations between time required to complete the 2-km walking test in 1997, and MetS score in 2021. Indeed, we observed a significant correlation (*r* = 0.211; *p* = 0.036) after adjusting for sex, age, SES, smoking, sleep quality and physical activity, indicating that a longer time to complete the 2-km walking test (reflective of worse cardiorespiratory fitness/ endurance) in 1997, is associated with higher MetS score in 2021. Thus, overall, there is evidence of an association between cardiorespiratory fitness assessed in 1997 and MetS in 2021 in our data, and the lack of an association between cardiorespiratory fitness assessed in 1992 and MetS in 2021 may be due to test-related/ methodological issues.

In further secondary analyses (please refer to supplementary Table 1), we compared the baseline characteristics of persons who participated in 1992 and 2021 (*N* = 89; sample of our current analysis) to the baseline characteristics of persons who only participated in 1992 and not in any further study assessments (*N* = 98, 50 females). We observed that persons who participated at baseline and at 29-years follow-up had significantly lower ages, higher SES (only true for females), higher sports related physical activity, higher fitness scores, as well as lower BMI and waist circumference at baseline. This may indicate a selective drop-out effect [31], i.e., mainly participants with higher fitness and who were potentially healthier at baseline also participated at 29-years follow-up and thus remained active study participants. This may also be a possible explanation of our observed associations between higher baseline fitness and lower MetS score at follow-up. Future research on fitness and health-related outcomes should aim at increasing variance in samples, and particularly encourage longitudinal study participation of individuals who are less inactive, or have lower fitness levels or health impairments.

Another explanation of our observed association between higher fitness and better metabolic health may be that fitness, as compared to physical activity behavior, remains rather stable over longer period of times. Indeed, we observed significant (*p* < 0.001) correlations between the fitness scores in 1992 and 2021 (*r* = 0.56 for males, and *r* = 0.72 for females). Nevertheless, as expected, fitness declines with increasing age in our sample, i.e., the fitness score among males decreased by about 15 z-values (corresponding to about 0.5 z-values per year) from 97.4 in 1992 to 82.5 in 2021, and from 93.9 to 79.1 for females, respectively. To put this into perspective, for example, a difference of 1 z-value in the sit-and-reach test among 40 years old individuals, corresponds to a difference of about 1 cm. Thus, the decrease in test performance between baseline and 29-years follow-up is about 5–10 cm, and resembles a decline in test performance of about 0.5–1% per year. Previous research (e.g., [32, 33]). has also reported that fitness as compared to physical activity tends to be rather stable over several years of follow-up. Similar to fitness, in our study, body mass index also remained rather stable of 29-years follow-up, with correlation coefficients of *r* = 0.73 for males, and *r* = 0.63 for females, and increases of 1.1 BMI points (corresponding to about 3.4 kg) for males, and 1.8 BMI points (corresponding to about 4.7 kg) for females between 1992 and 2021. To conclude, with regard to interpretation of our study findings, it is important to note that physical performance declines over the life span, at a rate of about 0.5 to 1% per year depending on the motor ability and performance level. It is further known that this decline accelerates with increasing age. Regular engagement in physical activity and maintaining a sufficient fitness level is thus particularly important in middle and late adulthood, and may decrease the risk of various health-related problems and disorders (including but not limited to MetS), as well as impairments in quality of life, well-being and independence with regard to activities of daily living.

The findings of our current study have implications for both clinical practice and future research endeavors. For example, clinicians, health counselors and exercise specialists should discuss and explain the importance of maintaining or improving fitness, particularly with patients or individuals at risk for metabolic disorders. Addressing the significance of fitness and motor performance in the clinical setting may also improve overall disease management and prevention of medical comorbidities.

Our study should be interpreted by considering its strengths and limitations. One strength is the long follow-up of 29 years, and the use of a validated, comprehensive test battery to assess fitness, including gross motor coordination, mobility/ flexibility, strength and cardiorespiratory fitness/ endurance [26]. Limitations pertain to the rather small sample size of only 89 participants, which also limited our ability to conduct further in-depth, stratified statistical analyses. For example, structural equation modeling or linear mixed effects regression models could provide a deeper understanding of the longitudinal relationships between fitness and metabolic risk factors. Also, as in any observational study, it is not possible to determine the direction of the association between fitness and MetS, and reverse causality may be possible. This means that either higher baseline fitness leads to better longitudinal metabolic health outcomes, or participants who eventually develop MetS during follow-up are less likely to engage in fitness-improving activities and exercises years or decades earlier, potentially due to underlying processes that occur in the body during incipient stages of metabolic health conditions. Furthermore, we did not examine potential mechanisms that explain the observed longitudinal association between fitness and MetS, except for the aforementioned potential selective drop-out effect and stability of fitness over time in our sample. However, it has been postulated in the literature that enhanced fitness may modulate the negative impact of MetS drivers such as diabetes-related insulin resistance, dysfunctional adipose fuel handling and central obesity [15]. In addition, we can speculate from the literature that fitness status serves as a mediator of the association between physical activity and metabolic syndrome. Physical activity, particularly exercise- or sports-related physical activity, is associated with various health outcomes, including but not limited to a reduced risk of metabolic syndrome. Physical activity can help to reduce blood pressure, reduce weight and improve lipid profiles, amongst other effects [34–36]. Furthermore, physical activity contributes to improved insulin sensitivity [37]. By definition, one of the main effects of physical activity is maintenance or improvement of fitness. These effects on fitness are mainly achieved if persons engage in physical activity on a regular basis and for prolonged periods of time. Thus, fitness status may be considered an even more valid predictor variable for determining health status, or metabolic health in particular, than physical activity. Another limitation of our study, as also mentioned above, is that our sample may be biased to the extent that physically active participants are more likely to engage in follow-up assessments than participants who are less physically active (selective drop out effect). This is also demonstrated by the high physical activity level of participants at follow-up. Nevertheless, this rather homogenous study sample with lower variance of physical activity and also fitness across participants may have actually reduced our power to detect associations between fitness and MetS. Yet, we were able to detect associations between fitness and MetS, and we can speculate that these associations may even be more pronounced in a more heterogeneous study sample with higher variation of physical activity and/ or fitness. Additionally, our study was conducted in a relatively small (about 13,000 inhabitants) and rather wealthy town in southwestern Germany, and study findings from this community may thus not be generalizable to overall Germany or even other countries. More research on the associations between fitness and metabolic health outcomes in geographically and ethnically diverse populations is thus warranted.

## Conclusion

In conclusion, our study builds on the growing body of research that provides evidence of a longitudinal association between fitness and metabolic health over a long follow-up period of 29 years. As such, fitness may also serve as a mediator of the previously reported longitudinal associations between physical activity and metabolic health. Our study highlights the importance of maintaining or improving fitness in adulthood, and clinicians or health practitioners may want to encourage patients at risk for MetS to engage in fitness-building activities or exercise. More research is needed to confirm our findings, and also to explore underlying mechanisms.

## Practical implications

- After controlling for age, sex, socioeconomic status, smoking, sleep quality and physical activity in min/ week, we observed a statistically significant correlation between higher fitness at baseline and lower metabolic syndrome score at 29-years follow-up.
- Fitness has important implications for healthy aging.
- Fitness may serve as a mediator on the associations between physical activity and metabolic health.

### Electronic supplementary material

Below is the link to the electronic supplementary material.

Supplementary Material 1

Supplementary Material 2

## Data Availability

The data that support the findings of this study are not openly available due to reasons of sensitivity and are available from the corresponding author upon reasonable request.

## Abbreviations

BMI: Body Mass Index
CR: Fitness Cardiorespiratory fitness
GM: Coordination Gross motor coordination
MetS: Metabolic Syndrome
PA: Physical Activity
SES: Socioeconomic Status
SQ: Sleep Quality
TC: Total cholesterol
TG: Triglycerides
