# Supplementary material for: Longitudinal association between fitness and metabolic syndrome: a population-based study over 29 years follow-up
Source: BMC Public Health. 2024 Apr 6;24:970. doi: 10.1186/s12889-024-18448-3 (PMC10998408; doi:10.1186/s12889-024-18448-3)
Supplement: Supplementary file 1 — Supplementary Material 1 [file 12889_2024_18448_MOESM1_ESM.docx]

*Supplementary table 1:* *Baseline characteristics of participants for longitudinal analysis (participation in 1992 and 2021) versus persons who only participated in 1992*

|  | 1992 (longitudinal) | | 1992 (only) | | t-test (p-value) | |
| --- | --- | --- | --- | --- | --- | --- |
|  | Females | Males | Females | Males | Females | Males |
|  | (N = 41) | (N = 48) | (N = 50) | (N = 48) |  |  |
| Age in years; mean (SD) | 38.7 (4.5) | 41.2 (6.3) | 46.9 (6.9) | 45.9 (7.5) | <.001 | .001 |
| BMI; mean (SD) | 23.3 (2.9) | 25.6 (2.8) | 26.1 (4.2) | 28.2 (4.0) | <.001 | <.001 |
| Fitness score (z-transformed); mean (SD) | 93.9 (5.3) | 97.4 (6.4) | 86.8 (6.2) | 93.9 (8.0) | <.001 | .019 |
| CR fitness/ endurance (z-transformed); mean (SD) | 84.7 (5.7) | 97.3 (8.3) | 84.4 (4.3) | 94.5 (8.6) | .816 | .167 |
| Strength (z-transformed); mean (SD) | 83.7 (6.2) | 98.4 (7.4) | 76.1 (6.7) | 93.5 (10.5) | <.001 | .013 |
| Flexibility/ mobility (z-transformed); mean (SD) | 106.7 (7.2) | 98.0 (9.9) | 99.3 (9.5) | 95.1 (12.3) | <.001 | .200 |
| GM coordination (z-transformed); mean (SD) | 97.9 (11.5) | 96.6 (9.2) | 85.1 (11.4) | 94.2 (12.7) | <.001 | .299 |
| Waist circumference in cm;mean (SD) | 77.9 (8.5) | 91.4 (9.3) | 85.6 (11.6) | 100.4 (11.6) | <.001 | <.001 |
| Blood glucose in mg/dl; mean (SD) | 86.7 (14.6) | 95.3 (21.9) | 87.2 (17.2) | 99.8 (49.9) | .892 | .571 |
| HDL-Cholesterol in mg/dl; mean (SD) | 65.0 (16.7) | 59.2 (13.9) | 65.1 (14.4) | 51.4 (10.2) | .976 | .003 |
| Systolic BP in mmHg; mean (SD) | 118.3 (11.5) | 130.7 (12.4) | 127.3 (13.6) | 135.7 (15.9) | .001 | .091 |
| Diastolic BP in mmHg; mean (SD) | 73.3 (8.3) | 78.8 (8.1) | 78.0 (8.3) | 80.9 (8.1) | .009 | .224 |
| SES score; mean (SD) | 2.9 (0.7) | 3.1 (0.9) | 2.4 (1.0) | 3.0 (1.1) | .022 | .680 |
| Sports-related PA in min/week; mean (SD) | 107.7 (112.9) | 113.9 (96.6) | 53.9 (74.2) | 53.5 (102.4) | .008 | .004 |
| General PA in min/ week; mean (SD) | 244.6 (179.7) | 343.9 (309.7) | 324.9 (264.8) | 395.3 (329.0) | .097 | .442 |

*SD, standard deviation; BMI, body mass index; CR fitness, cardiorespiratory fitness; GM coordination, gross motor coordination; MetS, metabolic syndrome, possible range 0-5; SES, socio-economic status, possible range 1-4; PA, self-reported physical activity; p, indicates statistical significance (derived from t-test)*
