# Supplementary material for: Longitudinal association between fitness and metabolic syndrome: a population-based study over 29 years follow-up
Source: BMC Public Health. 2024 Apr 6;24:970. doi: 10.1186/s12889-024-18448-3 (PMC10998408; doi:10.1186/s12889-024-18448-3)
Supplement: Supplementary file 2 — Supplementary Material 2 [file 12889_2024_18448_MOESM2_ESM.docx]

*Supplementary table 2: Summary of correlations on fitness and individual fitness components in 1992 and MetS score in 2021*

|  | Unadjusted | | Adjusted for age, sex and SES | | Additionally adjusted for smoking and sleep quality | | Additionally adjusted for PA | |
| --- | --- | --- | --- | --- | --- | --- | --- | --- |
|  | r | p | r | p | r | p | r | p |
| Fitness score | -0.251 | 0.022 | -0.212 | 0.059 | -0.291 | 0.011 | -0.304 | 0.009 |
| CR fitness/ endurance | -0.079 | 0.525 | -0.038 | 0.766 | -0.026 | 0.843 | -0.091 | 0.493 |
| Strength | -0.237 | 0.032 | -0.224 | 0.048 | -0.300 | 0.010 | -0.295 | 0.012 |
| GM coordination | -0.228 | 0.037 | -0.210 | 0.062 | -0.274 | 0.017 | -0.279 | 0.017 |
| Flexibility/ mobility | -0.023 | 0.837 | -0.038 | 0.739 | -0.045 | 0.699 | -0.044 | 0.709 |

*r, correlation coefficient; p, indicates statistical significance; CR fitness, cardiorespiratory fitness; GM coordination, gross motor coordination; MetS, metabolic syndrome; SES, socio-economic status; PA, self-reported physical activity*
